# Iodine Intake and Risk of Mortality: Evidence from a Nationally Representative Korean Cohort

**DOI:** 10.3390/nu17243859

**Published:** 2025-12-10

**Authors:** Jung-Hwan Cho, Jun Young Kim, Nak Gyeong Ko, Hanaro Park, Byung Soo Kwan, Ji Min Han, Sunghwan Suh, Ji Cheol Bae, Tae Hyuk Kim, Sun Wook Kim, Jae Hoon Chung, Hye Rang Bak, Hye In Kim

**Affiliations:** 1 Division of Endocrinology and Metabolism, Department of Internal Medicine, Samsung Changwon Hospital, Sungkyunkwan University School of Medicine, Changwon 51353, Republic of Korea; whwjdzl1004@gmail.com (J.-H.C.); jimin1981@naver.com (J.M.H.); sh23.suh@samsung.com (S.S.); drkuri10@gmail.com (J.C.B.); 2 Department of Internal Medicine, Samsung Changwon Hospital, Sungkyunkwan University School of Medicine, Changwon 51353, Republic of Koreakbs2459@naver.com (B.S.K.); 3 Department of Research and Support, Samsung Changwon Hospital, Sungkyunkwan University School of Medicine, Changwon 51353, Republic of Korea; nakgyeong.ko@samsung.com; 4 Department of Otorhinolaryngology-Head & Neck Surgery, Samsung Changwon Hospital, Sungkyunkwan University School of Medicine, Changwon 51353, Republic of Korea; naronaro1983@gmail.com; 5 Division of Endocrinology & Metabolism, Department of Internal Medicine, Thyroid Center, Samsung Medical Center, Sungkyunkwan University School of Medicine, Seoul 06351, Republic of Korea; taehyukmd.kim@samsung.com (T.H.K.); sunwooksmc.kim@samsung.com (S.W.K.); jaeh.chung@samsung.com (J.H.C.); 6 Department of Family Medicine, Samsung Changwon Hospital, Sungkyunkwan University School of Medicine, Changwon 51353, Republic of Korea

**Keywords:** micronutrients, iodine, urinary iodine concentration, mortality, Korean national health and nutrition examination survey, Asian

## Abstract

**Background/Objectives:** Iodine intake influences thyroid-related and metabolic diseases that have important public health implications. However, longitudinal evidence of the association between iodine intake and mortality remains scarce and limited to Western populations. Given the markedly high iodine intake among Asians and possible ethnic or regional differences in iodine sensitivity, population-based data from Asian cohorts are needed. **Methods:** We analyzed 5497 adults from the Korean National Health and Nutrition Examination Survey (2013–2015) linked with mortality follow-up through 2021. Urinary iodine concentration (UIC) was quantified from spot urine samples using inductively coupled plasma mass spectrometry. Iodine intake was estimated from UIC and categorized into four groups: below the estimated average requirement, low-normal, high-normal, and above the tolerable upper level (UL). The primary outcome was all-cause mortality. Cardiovascular disease-specific and cancer-specific mortality were also assessed. Multivariable Cox proportional hazards models accounting for the complex survey design were used to estimate hazard ratios and 95% confidence intervals. Sensitivity analysis excluded participants with thyroid disease or early death, and subgroup analyses by age and sex were also conducted. **Results:** During a median 8.4-year follow-up, 139 deaths occurred. Compared with the low-normal intake group, excessive iodine intake (above UL) was not associated with all-cause mortality (HR 1.09, 95% CI 0.36–3.27), cardiovascular mortality (HR 1.27, 95% CI 0.21–7.61), or cancer mortality (HR 1.71, 95% CI 0.40–7.29). No significant trends were observed across intake categories (*p* > 0.2), and similar findings were obtained when UIC levels were analyzed. Excluding participants with thyroid disease or early death did not change the results, and no significant interaction was observed by age or sex. **Conclusions:** In this first population-based Asian study on iodine intake to mortality, neither estimated iodine intake nor UIC was associated with all-cause mortality. These results suggest that the relationship between iodine exposure and mortality may differ across populations, underscoring the need for further large-scale studies.

## 1. Introduction

Iodine is a key micronutrient required for thyroid hormone synthesis, which regulates diverse metabolic processes in human physiology. Historically, deficiency rather than excess of iodine has been recognized as a major global health problem, leading to goiter and neurodevelopmental impairments [1,2].

Accordingly, national public health agencies, including the World Health Organization (WHO) and the Institute of Medicine (IOM), have implemented objective monitoring of population iodine status and have promoted iodine supplementation through public health interventions. At the population level, the WHO recommends urinary iodine concentration (UIC) measured from spot urine samples as the primary indicator of iodine status, given that more than 90% of ingested iodine is excreted in the urine within 24 h. Population iodine status is commonly categorized according to median UIC values, with levels below 100 μg/L indicating insufficiency, 100–199 μg/L adequacy, 200–299 μg/L above requirements, and ≥300 μg/L excess [3]. However, at the individual level, UIC may deviate from actual iodine intake depending on factors such as muscle mass, age, and sex. Therefore, to ensure adequate iodine nutrition, IOM emphasizes iodine intake rather than UIC itself, advising consumption above the estimated average requirement (EAR, 150 μg/day) while avoiding intake beyond the tolerable upper intake level (UL, 1100 μg/day) [4]. In addition, spot UIC exhibits substantial day-to-day variability depending on recent dietary intake and may not adequately reflect long-term iodine exposure. Regarding measurement, UIC is typically quantified using inductively coupled plasma mass spectrometry (ICP-MS), which provides high analytical sensitivity and precision for trace-level iodine measurement and has been widely adopted in national surveillance programs [2,3,4].

As iodization programs by both the IOM and WHO have expanded [3,4], however, many populations have moved from insufficiency toward sufficiency or even excess [5,6,7]. Therefore, questions have emerged about the broader health effects of varying iodine exposure [8,9,10]. Beyond its impact on thyroid function, iodine exposure may influence long-term health outcomes, including mortality, considering that both hypothyroidism and hyperthyroidism have been linked to cardiovascular and metabolic risk [11,12,13]. To minimize the risk of thyroid dysfunction related to iodine excess, national public health agencies have therefore established UL based on thyroid-related adverse outcomes from previous population-based data [4].

In contrast to Western countries [14,15], East Asia represents a distinct iodine-replete region due to habitual seaweed consumption [8,16,17]. According to the Korea National Health and Nutrition Examination Survey (KNHANES), the median UIC was close to 300 μg/L, and approximately 13.4% of adults consumed iodine above UL [17]. Despite these unique exposure characteristics, virtually all existing longitudinal evidence on iodine excess and long-term health outcomes has been confined to Western populations [14,15]. Given potential ethnic differences in thyroid set-points, genetic sensitivity, and dietary iodine sources, evidence from Asian cohorts is needed. Moreover, previous studies relied on UIC levels, which are less applicable for policy guidance. Estimating iodine intake from standardized UIC-based equations [17,18] may be a better exposure variable for public health interventions.

Therefore, we used nationally representative Korean survey data linked to mortality records to examine the association between estimated iodine intake (and corresponding UIC levels) and all-cause and cause-specific mortality.

## 2. Materials and Methods

### 2.1. Study Population

**Figure 1:**
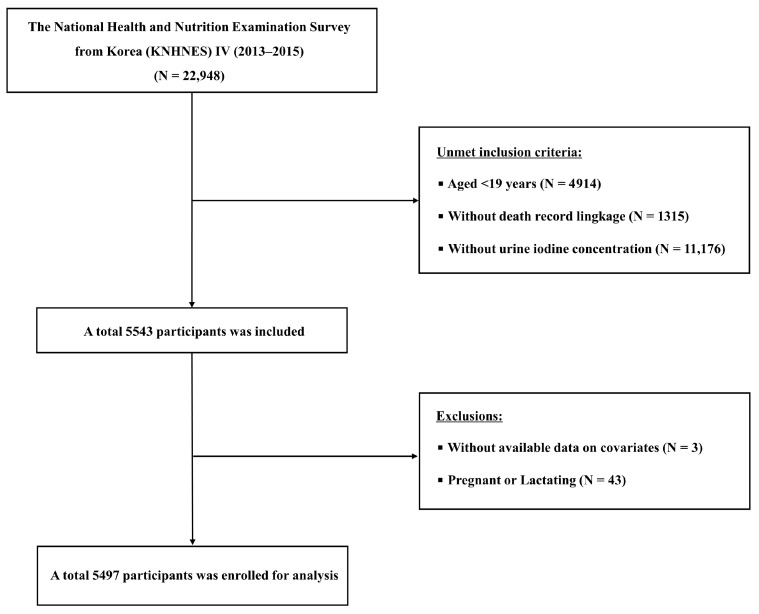
Flowchart of participant enrollment.

The Korea National Health and Nutrition Examination Survey (KNHANES) VI (2013–2015) was conducted using a nationally representative, stratified, multistage sampling framework designed to capture the general Korean population (*n* = 22,948). Participants underwent standardized health interviews and physical examinations, and a subset also provided blood and urine specimens. Study protocols were approved by the Institutional Review Board of the Korea Centers for Disease Control and Prevention, and written informed consent was obtained from all participants. Approximately one-third of the total sample was randomly selected for additional thyroid function testing, including serum thyroid-stimulating hormone (TSH), free thyroxine, and UIC levels. Mortality was prospectively ascertained through linkage to national mortality records up to December 2021 [19]. Of the 5543 adult participants with linked mortality data and available data for UIC, individuals without available data on covariates (*n* = 3) or who are pregnant or lactating (*n* = 43) were excluded. After these exclusions, 5497 participants were retained in the analysis cohort (Figure 1).

### 2.2. Variable Specifications

**UIC and estimated iodine intake**

UIC was determined from single spot urine samples using inductively coupled plasma mass spectrometry (ICP-MS; PerkinElmer, Waltham, MA, USA) (reference range 42–350 µg/L) with an iodine standard solution (Inorganic Venture, Christiansburg, VA, USA). This analytical method provides high sensitivity with a detection limit in the low μg/L range, long-term coefficients of variation of approximately 2–3% across low and high concentration ranges, and near-quantitative recovery in external quality-control urine materials [20].

Because a single UIC measurement may fluctuate due to day-to-day variation, it is considered more appropriate for evaluating population iodine status rather than individual exposure. To estimate iodine intake at the individual level and to facilitate dietary and mortality-related analyses, we used ‘estimated iodine intake’ as the main exposure variable. This variable was derived from a previously validated equation developed for the KNHANES cohort, incorporating age, sex, body weight, urinary creatinine concentration, and creatinine excretion rate. The detailed calculation formula is described as follows [17,18,21,22]:

Estimated iodine intake = UIC × {879.89 + (Body weight × 12.51) − {(6.19 × Age) + (34.51 if black) − (379.42 if women)}/(urine creatinine × 0.92 × 10)

The participants were divided into four categories of estimated iodine intake (µg/day) based on IOM [3]: below the estimated average requirement (EAR) (0–149), low normal (150–299), upper normal (300–1099), and above the tolerable upper intake level (UL) (≥1100). UIC levels (µg/L) were subclassified into five categories using WHO recommendations for population iodine status [4]: very low (0–49), low (50–99), adequate (100–299), high (300–399), and excessive (≥400).

**Outcomes**

All-cause mortality was selected as the primary endpoint because it represents the most definitive and clinically relevant outcome, integrating the cumulative effects of thyroid dysfunction and its associated cardiometabolic consequences. Cardiovascular disease (CVD)—and cancer-specific mortality were also assessed as secondary outcomes. Mortality status was identified through record linkage with the National Death Index using death certificate information provided by the Korea Disease Control and Prevention Agency. Cause of death was classified according to the International Classification of Diseases, 10th Revision, as follows: all-cause mortality (A00–Y98), CVD-specific mortality (I00–I99), and cancer-specific mortality (C00–C97).

**Other variables**

Sociodemographic and lifestyle information, including education level, household income, alcohol consumption, and smoking status, was obtained through standardized questionnaires. Educational attainment was categorized into three groups: middle school, high school, and university graduation. Monthly household income was reported in Korean won and classified into three levels (low, middle, and high).

Anthropometric measurements, laboratory tests, and dietary questionnaires were used to assess body mass index (BMI), estimated glomerular filtration rate (eGFR), underlying comorbidities, sodium intake, and the frequency of seaweed consumption. Comorbid conditions included diabetes mellitus (defined as a self-reported physician diagnosis, fasting glucose ≥126 mg/dL, or HbA1c ≥ 6.5%) [23], dyslipidemia (defined as a self-reported physician diagnosis, triglyceride ≥200 mg/dL, low-density lipoprotein cholesterol ≥ 160 mg/dL or high-density lipoprotein cholesterol <40 mg/dL) [24], hypertension (defined as a self-reported physician diagnosis, or ≥140/90 mmHg) [25], CVD (defined as a self-reported physician diagnosis), and any cancer history (defined as a self-reported physician diagnosis).

### 2.3. Statistical Analysis

Variables with normal distributions are presented as mean ± standard deviation and compared using analysis of variance. Non-normally distributed variables are presented as median (interquartile range, IQR) and compared using the Kruskal–Wallis test. Categorical variables are expressed as numbers (percentages) and compared using the chi-square test. Two-sided *p*-values of < 0.05 were considered statistically significant.

Descriptive statistics were generated using chi-square tests for categorical variables and analysis of variance for continuous variables. Crude and multivariable Cox proportional hazards regression models were applied to estimate hazard ratios (HRs) and 95% confidence intervals (CIs) for mortality outcomes. Model 1 was adjusted for age, sex, income, education, alcohol consumption, and smoking status, whereas Model 2 further included BMI, eGFR, sodium intake, and comorbidities (diabetes, dyslipidemia, hypertension, cardiovascular disease, and cancer). Individual food items were not included in the primary models because UIC already captures integrated dietary iodine exposure, and single-day food data are prone to substantial measurement error; sodium intake was adjusted, given its established cardiovascular relevance.

To verify the robustness of our findings, several sensitivity analyses were conducted. First, the analyses were repeated using raw UIC levels instead of estimated iodine intake. Second, we conducted the main analysis after excluding participants with a history of thyroid disease, or those who died within two years after baseline (lag-analysis) were excluded. Third, stratum-specific analyses were performed to evaluate potential effect modification by age (20–39, 40–59, and ≥60 years) and sex. All sensitivity or stratified analyses were performed using multivariable models adjusted for the same covariates as the primary analysis.

All statistical analyses were performed using STATA software (version 22.0, Armonk, NY, USA). Sampling weights were applied to account for the complex survey design, differential probabilities of selection, and nonresponse. Standard methods for descriptive comparisons and Cox proportional hazards regression were applied as previously described [26,27].

## 3. Results

### 3.1. Baseline Characteristics

The mean age was 41.3 ± 13.7 years, and 59.0% were men. The median UIC and estimated iodine intake were 275.1 µg/L (IQR 151.5–622.2) and 240.2 µg/day (IQR 129.4–552.6), respectively. The median estimated iodine intake levels in the below EAR, low normal, high normal, and above UL groups were 98.6 µg/day (74.3–123.4), 206.8 µg/day (177.5–246.7), 484.8 µg/day (371.4–677.9), and 2034.7 µg/day (1417.7–3362.7), respectively. Among participants in the above UL group, 326 of 774 individuals (42.1%) exceeded the Korean UL of 2400 µg/day. As expected, higher iodine intake levels were positively associated with frequency of seaweed soup (*p* = 0.006) or sea lettuce salad consumption (*p* = 0.001). Demographic, socioeconomic, metabolic parameters, and comorbidities except CVD differed significantly across estimated iodine intake groups. Higher iodine intake was associated with a higher income and educational level, as well as a higher prevalence of metabolic disorders and cancer (Table 1). Additional dietary details from the 24 h recall survey have been clarified in Supplementary Table S1.

### 3.2. Association Between Iodine Exposure and Mortality

In the multivariable Cox models using the low-normal intake group as the reference, estimated iodine intake was not significantly associated with all-cause or cause-specific mortality (Table 2). In the fully adjusted model (Model 2), the HRs (95% CIs) for excessive iodine intake (above UL) were 1.09 (0.36–3.27) for all-cause mortality, 1.12 (0.08, 15.09) for cardiovascular mortality, and 1.72 (0.36, 8.27) for cancer-specific mortality. No significant linear trend was observed across intake categories (all *p* for trend > 0.2).

When UIC was used instead of estimated intake, the results remained similar (Table 3). Fully adjusted HRs (95% CIs) for very high UIC (≥400 µg/L) were 0.69 (0.34, 1.40) for all-cause, 0.84 (0.14, 5.13) for cardiovascular, and 0.76 (0.29, 1.97) for cancer-specific mortality (all *p* for trend > 0.2).

### 3.3. Sensitivity and Lag-Time Analyses

When participants with thyroid disease at baseline were excluded (*n* = 4934), the association between excessive iodine intake (above UL) and mortality remained nonsignificant. The fully adjusted HRs (95% CIs) were 0.93 (0.27–3.26) for all-cause, 1.76 (0.23–13.65) for cardiovascular, and 0.60 (0.08–4.44) for cancer-specific mortality (all *p* for trend > 0.2) (Table 4).

In a lag analysis excluding deaths that occurred within the first 2 years of follow-up, the results were largely unchanged. Fully adjusted HRs (95% CIs) for excessive iodine intake were 1.26 (0.33–4.84) for all-cause mortality. Cancer-specific mortality was modestly elevated in the higher-intake groups in the crude {HR (95% CI) = 2.59 (1.15–5.80)} and Model 1 {HR (95% CI) = 3.13 (1.25–7.81)} analyses, but that association was no longer significant after full adjustment {HR (95% CI) = 2.69 (0.65–11.07)} (Table 5).

Overall, both sensitivity and lag analyses did not demonstrate a significant association between estimated iodine intake and mortality.

### 3.4. Stratified Analyses by Age and Sex

In age-stratified analyses using the low-normal iodine intake group as the reference, no significant associations were observed between excessive iodine intake and all-cause mortality (Supplementary Table S2). Among participants aged 20–39, 40–59, and ≥60 years, the fully adjusted HRs (95% CIs) for excessive iodine intake (above UL) were 0.75 (0.06–8.69), 1.76 (0.41–7.53), and 0.30 (0.02–3.89), respectively. When stratified by sex, the corresponding HRs (95% CIs) were 1.12 (0.32–3.96) for men and 2.17 (0.13–34.94) for women, showing no significant difference across subgroups.

## 4. Discussion

### 4.1. Principal Findings

Iodine intake was not clearly associated with all-cause or cause-specific mortality in this large, nationally representative Korean cohort. This first population-based study in Asia provided insight into the long-term health implications of iodine intake.

### 4.2. Comparison with Previous Studies

There is abundant evidence that both iodine deficiency [1,2] and excess [8,9,10] are linked to adverse health outcomes such as thyroid hormone production impairment, goiter, neurodevelopmental deficits in children, autoimmune thyroid disease, and thyroid cancer. Regarding mortality, however, only two longitudinal national cohorts [14,15] have examined urinary iodine and mortality, both from Western populations. In a Spanish community-based cohort of approximately 2000 adults followed for ten years [15], lower UIC (<100 µg/L) levels were associated with higher all-cause mortality (HR 1.71). Conversely, the U.S. NHANES III study, which included more than 12,000 adults with a 19-year follow-up period, reported a modest increase in all-cause mortality (HR 1.19) at excessive iodine levels (UIC > 400 µg/L), possibly reflecting thyrotoxicosis, or heightened cardiovascular stress induced by chronic iodine excess [14]. Taken together, the available evidence remains inconsistent, limited to Western populations, and has used simple UIC levels. In contrast, in the present Korean cohort, which represents the first population-based Asian data, iodine intake or UIC level was not significantly associated with mortality. In the fully adjusted model (Table 2), high iodine intake was not significantly associated with all-cause mortality, and this null association remained consistent across multiple sensitivity analyses (Table 3).

### 4.3. Biological and Dietary Explanations

Several biological and population-specific factors could explain this discrepancy. First, genetic differences could lead to the absence of the association between iodine intake and mortality in this Korean cohort. A recent study from Korea demonstrated that thyroid response to iodine exposure differed based on a polymorphism in the sodium–iodide symporter gene (*SLC5A5*, rs77277498) [28]. In addition to pre-existing genetic traits, generations of exposure to iodine-rich diets may favor alleles tolerant to chronic iodine excess. Consistently, East-Asian populations show higher TSH reference ranges than Western populations [29,30]. Such population-specific adaptations could act as a buffer against systemic impacts of further iodine excess. Second, differences in dietary iodine sources may also play a role. As shown in Table 1, participants with higher iodine intake were more likely to have not only higher sodium intake but also greater seaweed consumption. In Western populations, iodized salt is predominant [5,14], whereas seaweed is the major iodine source in East Asia [8,16]. Although the abovementioned U.S. study presented supplementary models adjusted for salt intake, residual confounding by salt consumption cannot be completely excluded. Apart from this, seaweed provides selenium, polyphenols, and fucoidan [31], which may mitigate oxidative or metabolic stress induced by excess iodine [32]. In line with this suggestion, a Japanese cohort study of 96,000 adults showed that almost-daily seaweed consumption was associated with approximately 20–30% lower cardiovascular mortality [33]. These findings imply that the seaweed sources of iodine may modulate the health impacts of excess intake compared to salt-based iodine.

Alternatively, structural and statistical factors may have contributed to the null findings. In the Korean cohort, the overall distribution of iodine exposure was skewed toward higher values, with 38.6% of participants in the very high UIC group (≥400 µg/L) compared with only 7.9% in the U.S. NHANES III [14]. Such a distribution limits the statistical sensitivity to detect a clear dose–response gradient. In addition, our cohort included 5497 participants and 139 deaths during a median 8.4-year follow-up period, whereas the NHANES III study comprised 12,264 participants and 3159 deaths over 19.2 years [14]. This smaller number of events and shorter observation period inevitably reduced the statistical precision to detect modest hazard ratios.

### 4.4. Strengths and Methodological Considerations

This study has several notable strengths with clinical implications. First, to our knowledge, the current study is the first nationally representative analysis in an Asian population evaluating the association between iodine exposure and mortality. Even though East Asia is one of the most iodine-replete regions worldwide, such evidence has been lacking. From a public-health perspective, these results suggest that excessive iodine exposure may not substantially affect overall survival in iodine-replete Asian populations, highlighting the need for population-specific studies when developing dietary guidelines for iodine intake. Second, iodine intake rather than simple UIC levels was provided as exposure in this study. Iodine intake, not UIC levels, which varied from even the same iodine intake by individual characteristics, is a modifiable factor. Public-health guidelines recommend an adequate range for iodine intake, not UIC levels [3,34]. Therefore, this study provides a more practical basis for nutritional policy. Third, multiple sensitivity analyses and adjustments for variables demonstrated the robustness of the findings.

### 4.5. Study Limitations and Future Prospects

Several limitations of this study should be acknowledged. First, the number of deaths was modest and the median follow-up relatively short, limiting the power to detect small-to-moderate effects. Although spot-urine iodine concentration is recommended by the WHO and the IOM for population monitoring, it may be influenced by recent dietary or contrast-media exposure and thus not sufficiently reflect long-term iodine status [18]. Future large-scale studies incorporating repeated urine measurements over time are warranted to confirm these results. Second, we should keep in mind that the current upper limits of iodine intake (1110 µg/day in IOM [3], 2400 µg/day in Korean Dietary Reference Intakes [34]) were derived in relation to thyroid disease (TSH elevation, goiter, or autoimmune thyroiditis) rather than mortality [35]. As the KNHANES–mortality linked dataset lacks longitudinal adjudication of thyroid dysfunctions or cardiovascular events, these intermediary outcomes could not be examined in the present study and remain an important topic for future research. Therefore, our findings should not be interpreted as evidence that excessive iodine intake is safe or change the current dietary guideline. Interestingly, a suggestive increase in cancer-specific mortality was observed in the lag-time analysis (Table 5), although this association was attenuated after full multivariable adjustment. While the primary analysis did not show a significant relationship between iodine intake and overall mortality, this finding highlights the need for further well-designed studies to clarify whether chronic iodine excess contributes to cancer mortality. Third, specific iodine-rich foods were not individually adjusted for, considering their role as upstream determinants of urinary iodine and the imprecision of single-day dietary recall; however, residual confounding by overall dietary patterns cannot be completely excluded.

Future studies incorporating repeated measurements of UIC are needed to better characterize long-term iodine exposure. In addition, longitudinal cohorts with adjudicated cardiovascular and cancer outcomes will be essential to clarify cause-specific pathways linking iodine excess to mortality. Further investigations focusing on genetic susceptibility and iodine sources, such as seaweed versus iodized salt, may also help explain population-specific differences in the health effects of iodine excess.

## 5. Conclusions

As the first population-based study in an Asian cohort, this analysis provides nationally representative evidence on the association between iodine exposure and mortality. Despite the high prevalence of excessive iodine intake in Korea, excessive iodine exposure was not associated with a substantial increase in overall mortality, suggesting that the health impact of chronic iodine excess may differ according to background iodine source or ethnicity. These findings underscore the need for large longitudinal studies across diverse populations to better define susceptible subgroups and clarify the long-term health consequences of sustained iodine excess. The potential associations with cardiovascular outcomes, however, cannot be excluded and warrant further investigation.

## Figures and Tables

**Table 1:** Baseline characteristics of the study population.

	Total (*n* = 5497)	Below EAR (*n* = 1654)	Low Normal (*n* = 1444)	High Normal (*n* = 1625)	Above UL (*n* = 774)	*p*- Value ^a^
Iodine levels						
Estimated iodine intake ^b^ (µg/day)	240.2 (129.4–552.6)	98.6 (74.3–123.4)	206.8 (177.5–246.7)	484.8 (371.4–677.9)	2034.7 (1417.7–3362.7)	<0.001
UIC levels (µg/L)	275.1 (151.5–622.2)	131.6 (87.6–187.7)	235.3 (161.3–329.9)	547.3 (354.0–855.7)	2070.0 (1235.3–3676.1)	<0.001
Frequency of seaweed intake (%)						
Seaweed soup ≥1/week	564 (13.5)	136 (10.6)	156 (14.0)	177 (14.8)	95 (16.9)	0.006
Sea lettuce salad ≥1/week	252 (6.2)	50 (3.7)	80 (7.6)	82 (7.0)	40 (7.2)	0.001
Total sodium intake (10^2^ mg/day)	34.8 ± 17.8	33.0 ± 16.6	35.3 ± 17.7	36.1 ± 18.6	35.1 ± 18.3	0.004
Age, *n* (%)	41.3 ± 14.1	38.6 ± 14.6	40.9 ± 13.1	42.8 ± 13.6	45.1 ± 14.4	<0.001
Men, *n* (%)	2814 (59.0)	746 (53.4)	801 (63.0)	867 (60.8)	400 (60.2)	<0.001
Income levels, *n* (%)						<0.001
Low	1805 (29.4)	605 (33.3)	451 (27.9)	515 (28.4)	234 (25.7)	
Middle	1809 (34.5)	542 (34.1)	509 (36.9)	527 (34.1)	231 (31.2)	
High	1859 (35.9)	502 (32.4)	481 (35.0)	575 (37.3)	301 (43.0)	
Education level, *n* (%)						0.024
Middle school	1177 (16.7)	374 (17.2)	284 (15.1)	328 (15.9)	191 (20.1)	
High school	1493 (28.3)	434 (26.4)	384 (27.7)	465 (30.1)	210 (30.0)	
University	2464 (54.9)	758 (56.2)	679 (57.1)	713 (53.8)	314 (49.7)	
Alcohol consumption, *n* (%)						0.001
<1/month	1713 (31.8)	569 (36.7)	397 (27.9)	495 (30.2)	252 (31.7)	
1~4/month	1951 (41.8)	570 (39.9)	547 (43.4)	579 (42.1)	255 (42.3)	
≥1/month	1210 (26.3)	328 (23.3)	347 (28.5)	375 (27.6)	160 (25.8)	
Smoking status, *n* (%)						0.004
Current smoker	1263 (27.5)	411 (28.2)	358 (29.2)	347 (26.1)	147 (25.2)	
Ex-smoker	1088 (21.0)	260 (17.5)	280 (20.2)	365 (24.1)	183 (24.3)	
Never-smoker	2949 (51.3)	934 (54.1)	749 (50.4)	850 (49.7)	416 (50.4)	
BMI (kg/m^2^)	23.8 ± 3.6	23.4 ± 3.7	23.9 ± 3.4	24.1 ± 3.5	24.1 ± 3.6	<0.001
eGFR (mL/min/1.73 m^2^)	100.0 ± 15.2	101.0 ± 14.8	100.6 ± 14.8	99.1 ± 15.8	98.3 ± 15.8	<0.001
Comorbidity, *n* (%)						
Diabetes	638 (9.6)	167 (7.7)	174 (10.3)	192 (9.9)	105 (11.8)	0.016
Dyslipidemia	1936 (33.6)	507 (29.3)	508 (33.1)	637 (38.3)	284 (34.6)	<0.001
Hypertension	1300 (20.1)	350 (17.1)	326 (19.3)	399 (21.5)	225 (25.4)	<0.001
CVD	147 (1.8)	44 (1.9)	35 (1.6)	43 (1.8)	25 (1.8)	0.955
Cancer	160 (2.2)	36 (1.4)	30 (1.6)	56 (2.8)	38 (3.8)	<0.001

EAR, estimated average requirement; UL, tolerable upper intake level; UIC, urine iodine concentration; BMI, body mass index; eGFR, estimated glomerular filtration rate; CVD, cardiovascular disease. Data are expressed as mean ± standard deviation for normally distributed variables or median (IQR) for skewed variables, and as number (%) for categorical variables. ^a^ *p*-values were obtained using ANOVA, Kruskal–Wallis, or chi-square tests as appropriate. ^b^ Estimated iodine intake = UIC × {879.89 + (Body weight × 12.51) − {(6.19 × Age) + (34.51 if black) − (379.42 if women)}/(urine creatinine × 0.92 × 10).

**Table 2:** HRs for all-cause and cause-specific mortality according to estimated iodine intake.

	Events/Participants (%)	Unadjusted HR (95% CI)	Model 1 ^a^ HR (95% CI)	Model 2 ^b^ HR (95% CI)
**All-cause mortality**				
Below EAR	41/1654	0.91 (0.54, 1.52)	0.94 (0.53, 1.68)	1.13 (0.47, 2.72)
Low normal	38/1444	reference	reference	reference
High normal	40/1625	1.16 (0.68, 1.99)	1.15 (0.61, 2.17)	1.43 (0.58, 3.51)
Above UL	20/774	0.94 (0.49, 1.80)	0.67 (0.31, 1.45)	1.09 (0.36, 3.27)
*p for trends*		0.567	0.752	0.746
**CVD-specific mortality**				
Below EAR	4/1654	0.46 (0.13, 1.63)	0.36 (0.10, 1.35)	0.60 (0.08, 4.59)
Low normal	8/1444	reference	reference	reference
High normal	3/1625	0.50 (0.12, 2.08)	0.42 (0.08, 2.14)	0.86 (0.09, 7.85)
Above UL	5/774	0.75 (0.21, 2.72)	0.32 (0.05, 1.95)	1.12 (0.08, 15.09)
*p for trends*		0.783	0.776	0.671
**Cancer-specific mortality**				
Below EAR	20/1654	1.16 (0.51, 2.61)	1.15 (0.44, 2.99)	1.87 (0.61, 5.68)
Low normal	16/1444	reference	reference	reference
High normal	25/1625	1.74 (0.79, 3.85)	1.87 (0.71, 4.91)	2.08 (0.65, 6.72)
Above UL	11/774	1.31 (0.52, 3.30)	1.02 (0.33, 3.13)	1.72 (0.36, 8.27)
*p for trends*		0.346	0.602	0.822

HR, hazard ratio; CI, confidence interval; EAR, estimated average requirement; UL, tolerable upper intake level; CVD, cardiovascular disease. ^a^ Adjusted for age, sex, income level, education level, alcohol consumption, and smoking status. ^b^ Adjusted for Model 1 + body mass index, estimated glomerular filtration rate, diabetes, dyslipidemia, hypertension, cardiovascular disease, cancer, and sodium intake.

**Table 3:** HRs for all-cause and cause-specific mortality according to simple UIC levels.

	Events/Participants (%)	Unadjusted HR (95% CI)	Model 1 ^a^ HR (95% CI)	Model 2 ^b^ HR (95% CI)
**All-cause mortality**				
Very low	3/164	0.55 (0.16, 1.91)	0.29 (0.06, 1.30)	-
Low	19/542	1.34 (0.73, 2.49)	0.92 (0.46, 1.85)	0.41 (0.13, 1.28)
Normal	58/2182	reference	reference	reference
High	10/486	0.91 (0.44, 1.88)	1.21 (0.56, 2.61)	0.35 (0.08, 1.45)
Very high	49/2123	0.89 (0.56, 1.40)	0.83 (0.48, 1.45)	0.69 (0.34, 1.40)
*p for trends*		0.482	0.991	0.757
**CVD-specific mortality**				
Very low	0/164	-	-	-
Low	0/542	-	-	-
Normal	10/2182	reference	reference	reference
High	1/486	0.31 (0.04, 2.56)	-	-
Very high	9/2123	0.76 (0.26, 2.18)	0.50 (0.14, 1.83)	0.84 (0.14, 5.13)
*p for trends*		0.583	0.774	0.506
**Cancer-specific mortality**				
Very low	0/164	-	-	-
Low	9/542	1.12 (0.45, 2.81)	0.42 (0.14, 1.25)	**0.09 (0.01, 0.82)** ^†^
Normal	29/2182	reference	reference	reference
High	5/486	1.12 (0.37, 3.34)	1.68 (0.54, 5.23)	-
Very high	29/2123	1.07 (0.56, 2.05)	1.12 (0.51, 2.45)	0.76 (0.29, 1.97)
*p for trends*		0.539	0.230	0.391

HR, hazard ratio; CI, confidence interval; CVD, cardiovascular disease. ^a^ Adjusted for age, sex, income level, education level, alcohol consumption, and smoking status ^b^ Adjusted for Model 1 + body mass index, estimated glomerular filtration rate, diabetes, dyslipidemia, hypertension, cardiovascular disease, cancer, and sodium intake. ^†^ statistical significance (*p* < 0.05).

**Table 4:** HRs for all-cause and cause-specific mortality according to estimated iodine intake (excluding participants with thyroid disease at baseline).

	Events/Participants (%)	Unadjusted HR (95% CI)	Model 1 ^a^ HR (95% CI)	Model 2 ^b^ HR (95% CI)
**All-cause mortality**				
Below EAR	37/1529	0.88 (0.51, 1.52)	0.89 (0.48, 1.65)	1.17 (0.46, 3.01)
Low normal	34/1316	reference	reference	reference
High normal	37/1439	1.29 (0.74, 2.25)	1.23 (0.63, 2.41)	1.88 (0.74, 4.79)
Above UL	15/650	0.80 (0.39, 1.63)	0.58 (0.25, 1.34)	0.93 (0.27, 3.26)
*p for trends*		0.510	0.817	0.699
**CVD-specific mortality**				
Below EAR	3/1529	0.37 (0.09, 1.57)	0.23 (0.04, 1.14)	0.57 (0.04, 8.68)
Low normal	7/1316	reference	reference	reference
High normal	3/1439	0.61 (0.14, 2.69)	0.43 (0.07, 2.50)	1.13 (0.08, 15.84)
Above UL	4/650	0.79 (0.19, 3.35)	0.34 (0.05, 2.42)	1.76 (0.23, 13.68)
*p for trends*		0.484	0.788	0.577
**Cancer-specific mortality**				
Below EAR	18/1529	1.00 (0.43, 2.32)	0.93 (0.35, 2.50)	1.41 (0.43, 4.63)
Low normal	16/1316	reference	reference	reference
High normal	22/1439	1.64 (0.72, 3.70)	1.70 (0.63, 4.55)	1.66 (0.50, 5.44)
Above UL	8/650	0.95 (0.35, 2.61)	0.63 (0.19, 2.07)	0.60 (0.08, 4.44)
*p for trends*		0.444	0.777	0.634

HR, hazard ratio; CI, confidence interval; EAR, estimated average requirement; UL, tolerable upper intake level; CVD, cardiovascular disease. ^a^ Adjusted for age, sex, income level, education level, alcohol consumption, and smoking status. ^b^ Adjusted for Model 1 + body mass index, estimated glomerular filtration rate, diabetes, dyslipidemia, hypertension, cardiovascular disease, cancer, and sodium intake.

**Table 5:** HRs for all-cause and cause-specific mortality according to estimated iodine intake (extending lag time for the first 2 years).

	Events/Participants (%)	Unadjusted HR (95% CI)	Model 1 ^a^ HR (95% CI)	Model 2 ^b^ HR (95% CI)
**All-cause mortality**				
Below EAR	31/1644	0.99 (0.54, 1.81)	1.17 (0.60, 2.28)	1.66 (0.49, 5.60)
Low normal	29/1435	reference	reference	reference
High normal	35/1620	1.43 (0.78, 2.62)	1.49 (0.74, 3.00)	1.96 (0.61, 6.28)
Above UL	17/771	1.08 (0.52, 2.24)	0.77 (0.33, 1.77)	1.26 (0.33, 4.84)
*p for trends*		0.589	0.874	0.568
**CVD-specific mortality**				
Below EAR	3/1653	0.66 (0.11, 3.86)	0.65 (0.14, 3.06)	-
Low normal	3/1439	reference	reference	reference
High normal	3/1625	1.03 (0.17, 6.31)	1.38 (0.21, 8.89)	-
Above UL	4/773	1.29 (0.22, 7.52)	0.93 (0.11, 7.52)	-
*p for trends*		0.589	0.983	-
**Cancer-specific mortality**				
Below EAR	14/1648	1.50 (0.63, 3.57)	1.75 (0.66, 4.63)	3.07 (0.77, 12.22)
Low normal	13/1441	reference	reference	reference
High normal	22/1622	**2.59 (1.15, 5.80)** ^†^	**3.13 (1.25, 7.81)** ^†^	2.69 (0.65, 11.07)
Above UL	9/772	1.50 (0.57, 4.01)	1.18 (0.39, 3.61)	1.29 (0.20, 8.12)
*p for trends*		0.330	0.616	0.658

HR, hazard ratio; CI, confidence interval; EAR, estimated average requirement; UL, tolerable upper intake level; CVD, cardiovascular disease. ^a^ Adjusted for age, sex, income level, education level, alcohol consumption, and smoking status. ^b^ Adjusted for Model 1 + body mass index, estimated glomerular filtration rate, diabetes, dyslipidemia, hypertension, cardiovascular disease, cancer, and sodium intake. ^†^ Statistical significance (*p* < 0.05).

## Data Availability

The datasets generated and/or analyzed during the current study are publicly available from the Korea National Health and Nutrition Examination Survey (KNHANES) conducted by the Korea Disease Control and Prevention Agency (KDCA), upon approval of a data access request. All data are fully anonymized.

## Supplementary Materials

The following supporting information can be downloaded at: https://www.mdpi.com/article/10.3390/nu17243859/s1, Table S1: Dietary intake of iodine-rich foods and macronutrients according to iodine intake categories; Table S2: HRs for all-cause mortality according to estimated iodine intake stratified by age and sex.

## Abbreviations

The following abbreviations are used in this manuscript:

UIC	Urinary Iodine Concentration
UL	tolerable Upper intake Level
HR	Hazard Ratio
CI	Confidence Interval
IOM	Institute of Medicine
WHO	World Health Organization
NHANES	National Health and Nutrition Examination Survey
KNHANES	Korea National Health and Nutrition Examination Survey
TSH	Thyroid-Stimulating Hormone
EAR	Estimated Average Requirement
CVD	Cardiovascular Disease
BMI	Body Mass Index
eGFR	estimated Glomerular Filtration Rate
IQR	Interquartile Range
