# Supplementary material for: Iodine Intake and Risk of Mortality: Evidence from a Nationally Representative Korean Cohort
Source: Nutrients. 2025 Dec 10;17(24):3859. doi: 10.3390/nu17243859 (PMC12735466; doi:10.3390/nu17243859)
Supplement: Supplementary file 1 [file nutrients-17-03859-s001.zip › nutrients-4029755-supplementary.pdf]

**Supplementary Table S1.** Dietary intake of iodine-rich foods and macronutrients according to iodine intake categories

| | Total (n = 5,497) | Below EAR (n = 1,654) | Low normal (n = 1,444) | High normal (n = 1,625) | Above UL (n = 774) | P-value[a] |
|---|---|---|---|---|---|---|
| Iodine levels | | | | | | |
| Estimated iodine intake[b] (μg/day) | 240.2 (129.4- 552.6) | 98.6 (74.3-123.4) | 206.8 (177.5-246.7) | 484.8 (371.4-677.9) | 2034.7 (1417.7- 3362.7) | <0.001 |
| UIC levels (μg/L) | 275.1 (151.5- 622.2) | 131.6 (87.6-187.7) | 235.3 (161.3-329.9) | 547.3 (354.0-855.7) | 2070.0 (1235.3- 3676.1) | <0.001 |
| Energy intake | 2166.1 ± 860.9 | 2107.1 ± 842.2 | 2199.6 ± 866.1 | 2209.2 ± 877.0 | 2143.8 ± 848.4 | 0.058 |
| Fat intake | 45.0 ± 26.9 | 45.1 ± 26.2 | 45.6 ± 26.8 | 45.7 ± 28.0 | 41.9 ± 26.2 | 0.079 |
| Protein intake | 71.1 ± 33.9 | 69.1 ± 32.9 | 72.2 ± 34.5 | 72.9 ± 34.3 | 69.8 ± 34.1 | 0.090 |
| Carbohydrate intake | 332.0 ± 116.2 | 321.4 ± 111.6 | 334.1 ± 118.5 | 339.4 ± 116.5 | 337.1 ± 119.8 | 0.008 |
| Total sodium intake ($10^2$ mg/day) | 34.8 ± 17.8 | 33.0 ± 16.6 | 35.3 ± 17.7 | 36.1 ± 18.6 | 35.1 ± 18.3 | 0.004 |
| Frequency of seaweed (%) | | | | | | |
| Seaweed soup ≥1/week | 564 (13.5) | 136 (10.6) | 156 (14.0) | 177 (14.8) | 95 (16.9) | 0.036 |
| Sea lettuce salad ≥1/week | 252 (6.2) | 50 (3.7) | 80 (7.6) | 82 (7.0) | 40 (7.2) | 0.013 |
| Roasted laver ≥1/week | 2489 (62.3) | 712 (59.6) | 683 (62.9) | 737 (63.0) | 357 (65.9) | 0.158 |
| Frequency of soups (%) | | | | | | |
| Korean Beef Soup ≥1/week | 366 (9.2) | 92 (6.8) | 104 (10.3) | 123 (10.9) | 47 (9.2) | 0.013 |
| Soybean Paste Soup ≥1/week | 1327 (31.8) | 368 (28.8) | 346 (31.1) | 402 (32.8) | 211 (38.0) | 0.010 |
| Kimchi stew ≥1/week | 1603 (42.4) | 495 (42.4) | 438 (43.2) | 462 (42.8) | 208 (39.9) | 0.751 |
| Frequency of grilled fish (%) | | | | | | 0.024 |
| Mackerel ≥1/week | 552 (13.6) | 146 (11.8) | 141 (12.8) | 174 (14.8) | 91 (17.2) | 0.043 |
| Largehead hairtail ≥1/week | 361 (8.5) | 87 (6.7) | 93 (7.7) | 119 (10.5) | 62 (10.6) | 0.009 |
| Anchovies ≥1/week | 1738 (41.8) | 472 (38.2) | 477 (42.2) | 541 (44.5) | 248 (43.9) | 0.046 |

EAR, estimated average requirement; UL, tolerable upper intake level; UIC, urine iodine concentration; Ucr, Urine creatinine.

[a]P-values were obtained using ANOVA, Kruskal–Wallis, or chi-square tests as appropriate. Data are expressed as mean ± standard deviation for normally distributed variables or median (IQR) for skewed variables, and as number (%) for categorical variables.

[b]Estimated iodine intake =UIC × {879.89 + (Body weight x 12.51) − [(6.19 × Age) + (34.51 if black) − (379.42 if women)]}/(Ucr × 0.92×10)

**Supplementary Table S2.** HRs for all-cause mortality according to estimated iodine intake stratified by age and sex

| | Events / participants (%) | Unadjusted HR (95% CI) | Model 1 [a] HR (95% CI) | Model 2 [b] HR (95% CI) |
|---|---|---|---|---|
| *Age groups* | | | | |
| **Age 20-39 (n = 2,107)** | | | | |
| Below EAR | 1 / 732 | 0.34 (0.03, 3.91) | 0.30 (0.03, 3.32) | 0.11 (0.01, 1.86) |
| Low normal | 2 / 581 | reference | reference | reference |
| High normal | 5 / 570 | 2.27 (0.41, 12.45) | 1.97 (0.38, 10.30) | 0.75 (0.06, 8.69) |
| Above UL | 0 / 224 | - | - | - |
| **Age 30-59 (n = 2,291)** | | | | |
| Below EAR | 11 / 614 | 0.65 (0.26, 1.61) | 0.87 (0.34, 2.26) | 1.31 (0.44, 3.88) |
| Low normal | 14 / 623 | reference | reference | reference |
| High normal | 15 / 735 | 0.91 (0.39, 2.12) | 1.22 (0.45, 3.30) | 1.89 (0.63, 5.65) |
| Above UL | 8 / 319 | 1.04 (0.41, 2.60) | 1.15 (0.39, 3.35) | 1.76 (0.41, 7.53) |
| **Age ≥60 (n = 1,099)** | | | | |
| Below EAR | 29 / 308 | 1.33 (0.72, 2.43) | 1.32 (0.64, 2.74) | 1.53 (0.26, 8.92) |
| Low normal | 22 / 240 | reference | reference | reference |
| High normal | 20 / 320 | 0.93 (0.47, 1.82) | 1.00 (0.46, 2.18) | 0.92 (0.08, 10.01) |
| Above UL | 12 / 231 | 0.49 (0.21, 1.14) | 0.40 (0.15, 1.04) | 0.30 (0.02, 3.89) |
| *Sex* | | | | |
| **Men (n = 2,814)** | | | | |
| Below EAR | 29 / 746 | 1.04 (0.55, 1.95) | 0.92 (0.47, 1.77) | 1.17 (0.42, 3.22) |
| Low normal | 28 / 801 | reference | reference | reference |
| High normal | 33 / 867 | 1.31 (0.71, 2.41) | 1.24 (0.63, 2.44) | 1.67 (0.62, 4.50) |
| Above UL | 17 / 400 | 1.06 (0.51, 2.20) | 0.70 (0.31, 1.60) | 1.12 (0.32, 3.96) |
| **Women (n = 2,683)** | | | | |
| Below EAR | 12 / 908 | 0.79 (0.29, 2.12) | 1.07 (0.29, 3.98) | 1.36 (0.18, 10.55) |
| Low normal | 10 / 643 | reference | reference | reference |
| High normal | 7 / 758 | 0.78 (0.25, 2.43) | 0.71 (0.14, 3.65) | 1.10 (0.06, 19.48) |
| Above UL | 3 / 374 | 0.66 (0.16, 2.69) | 0.61 (0.10, 3.81) | 2.17 (0.13, 34.94) |

HR, hazard ratio; CI, confidence interval; EAR, estimated average requirement; UL, tolerable upper intake level.

[a] Adjusted for age, sex, income level, education level, alcohol consumption, and smoking status

[b] Adjusted for Model 1 + body mass index, estimated glomerular filtration rate, diabetes, dyslipidemia, hypertension, cardiovascular disease, cancer, and sodium intake
